# Urinary IL-18 is associated with arterial stiffness in patients with type 2 diabetes

**DOI:** 10.3389/fendo.2022.956186

**Published:** 2022-10-03

**Authors:** Caifeng Shi, Aiqin He, Xiaomei Wu, Lulu Wang, Xueting Zhu, Lei Jiang, Junwei Yang, Yang Zhou

**Affiliations:** Center for Kidney Disease, Second Affiliated Hospital of Nanjing Medical University, Nanjing, China

**Keywords:** IL-18, pulse wave velocity, arterial stiffness, diabetic kidney disease, inflammation

## Abstract

**Objective:**

Diabetic kidney disease (DKD) has been shown to be associated with an excess risk of cardiovascular death. Inflammation has been considered central to type 2 diabetes (T2D) pathophysiology, and inflammation markers have been linked to cardiovascular disease. The serum and urinary IL-18 levels were significantly elevated in patients with T2D; however, whether interleukin 18 (IL-18) are associated with the severity of arterial stiffness remains to be determined. This study examined the relationship of IL-18 levels with pulse wave velocity (PWV) as a reflector for arterial stiffness in patients with T2D.

**Methods:**

A total of 180 participants with T2D who had undergone PWV examination were enrolled. Serum and urinary IL-18 levels were measured using sandwich enzyme linked immunosorbent assay (ELISA) kits. Arterial stiffness was determined by carotid–femoral PWV (cf-PWV) and carotid–radial PWV (cr-PWV).

**Results:**

The urinary IL-18 levels correlated positively with cf-PWV in patients with T2D with DKD (*r* = 0.418, *p* < 0.001); however, we found no significant correlation between urinary IL-18 and cf-PWV in diabetic subjects without DKD. In addition, we found no significant correlation between urinary IL-18 and cr-PWV in participants with T2D with or without DKD. Moreover, the association remained significant when controlling for arterial stiffness risk factors, urinary albumin-to-creatinine ratio and estimated glomerular filtration rate. cf-PWV was greater in the higher group of urinary IL-18 than in the lower group. Nevertheless, we found no significant correlation between serum IL-18 and cf-PWV in participants with T2D.

**Conclusion:**

The urinary IL-18 levels appear to be associated with greater cf-PWV, suggesting the link between urinary IL-18 and arterial stiffness in patients with T2D.

## Introduction

The association between cardiovascular disease (CVD) and diabetes has been known for decades. Clinical and experimental studies have provided evidence and probable mechanisms that link diabetes to increased atherosclerosis. In particular, patients with T2D had more extensive atherosclerotic CVD (ASCVD) and more non-calcified plaques than patients with T1D. Further experimental studies are needed to elucidate these questions (1). Greater calcification in arterial media is also found in patients with diabetes compared with control subjects, especially T2DM (2). Diabetic kidney disease (DKD) has been shown to be associated with an excess risk of premature death and CVD death (3, 4). Despite this, the reasons for this relationship are incompletely understood.

The relationship of inflammation to insulin resistance is considered central to T2DM pathophysiology (5, 6). Markers of inflammation have been linked to CVD and CVD death. Clinical trials have overwhelmingly shown beneficial effect of targeting inflammation in prevention of the incidence of CVD in human with diabetes (7, 8). Moreover, systemic inhibition of nucleotide-binding oligomerization domain-like receptor thermal protein domain associated protein 3 (NLRP3) inflammasome was recently described to prevent increased atherosclerosis in mice with diabetes (9). IL-18 is a proinflammatory marker (10) and biomarker of kidney tubule injury and repair (11, 12). The serum and urinary IL-18 levels were significantly elevated in patients with T2D compared with control subjects (13). Elevated serum levels of IL-18 were associated with carotid intima-media thickness (13) and development of DKD in normal albuminuria subjects (14).

Unlike renal and retinal microvascular disease, there is no pathological fingerprint identifying a distinct atherosclerosis or arterial media calcification in the setting of diabetes. Pulse wave velocity (PWV) is assessed by measuring transit distance and transit time between two sites in the arterial system and taking their ratio. Carotid–femoral PWV (cf-PWV) is the current clinical gold standard measurement of arterial stiffness and has been established as a cardiovascular risk marker (15).

The aim of this study was to examine the relationship of IL-18 levels with PWV as a reflector for arterial stiffness and CVD.

## Methods

### Subjects

The study protocol was approved by Institutional Ethical Committee of Nanjing Medical University (approved No. 2019KY097), and written informed consent was obtained from all subjects. The study conforms to the principles outlined in the Declaration of Helsinki. A total of 180 subjects for this study were enrolled from patients with T2D of the Department of Internal Medicine at the Second Affiliated Hospital of Nanjing Medical University, who had undergone PWV examination between January 2020 and December 2020. Subjects were diagnosed as having T2D according to the WHO criteria and provided multiple morning urine and blood samples for assessment of urinary albumin-to-creatinine ratio (UACR) and estimated glomerular filtration rate (eGFR). On the basis of multiple UACR and eGFR measurements, subjects were classified as T2D without DKD (T2D − DKD, n = 115) or T2D with DKD (T2D + DKD, n = 65). Subjects with acute inflammatory diseases or malignant neoplasm were excluded, because the levels of inflammation can be markedly enhanced by such disease. Hypertension was defined as a blood pressure (BP) ≥ 130/80 mmHg or current use of antihypertensive medications.

### Blood and urine examination

Each individual provided blood and morning spot urine samples at baseline for biochemical measurements. Samples were centrifuged at 3,000 rpm at 4°C for 15 min, and aliquots were stored at −80°C if not analyzed immediately. Serum and urinary IL-18 levels were measured using sandwich ELISA kits (DY318-05, R&D Systems). Urinary IL-18 was normalized by urinary creatinine (UCr). The intra- and inter-assay coefficient of variations (CVs) for IL-18 were both less than 10%. The sensitivity of the assay was 5.47 pg/ml. These parameters were measured twice for each individual, and geometric mean was used as the baseline value in the analysis.

### Measurement of pulse wave velocity

cf-PWV and carotid–radial PWV (cr-PWV) were determined on a fasting state in the morning under room temperature (21°C–25°C) using the Complior Analyzer device (Artech Medical, Paris, France) according to the manufacturer’s introduction. Coffee, tea, or nitrates were not allowed within 2 h, and long-acting nitrates were restrained for 12 h before measurement. An experienced technician from the Second Affiliated Hospital of Nanjing Medical University performed the test for all participants. Before measurement, patients rested for 10 min and had their BP measured using a validated oscillometric device (Omron HEM-7130: Omron Healthcare Co., Ltd., Kyoto, Japan). Three probes were placed in a place of palpable pulse of the carotid, femoral, and radial artery, respectively. Ten consecutive recordings were averaged to calculate the transit time using the intersecting tangent algorithm. Carotid–femoral and carotid–radial distances were calculated as direct measurements multiplied by 0.8. Any measurement with a tolerance value more than 3 ms was considered invalid.

### Statistical analysis

All analyses were performed with SPSS 25 software package. Because the distribution of the IL-18 levels appeared to be left-skewed, they were normalized by log-transformation. Comparisons between groups were performed by using an unpaired Student’s *t*-test for normally distributed variables and a Mann–Whitney *U*-test for non-normally distributed variables. Associations between IL-18 levels and characteristics of type 2 diabetes were examined by Pearson correlation analysis for continuous variables and by Spearman correlation test for categorical variables. Association between urinary IL-18 and cf-PWV was determined by a multivariable linear regression analysis with a stepwise backward method. Mean cf-PWV was compared across the median of the urinary IL-18 levels by the general linear model, followed by covariance analysis. A *p*-value of less than 0.05 was taken to be statistically significant.

## Results

Clinical characteristics in **Table 1** indicate that participants with type 2 diabetes had a median age of 56.00 (47.00, 60.00) years, and 66.1% were male patients. Furthermore, participants had mean body mass index (BMI) of 25.01 (22.95, 27.52) kg/m^2^, HbA1c level of 8.30% (7.00%, 10.23%), and eGFR of 101.77 (92.47, 109.48) ml/min/1.73 m^2^. A proportion had albuminuria (35.6%) and hypertension (40%).

**Table 1:** Clinical characteristics of subjects with type 2 diabetes.

	Total (n = 180)	T2D − DKD (n = 115)	T2D + DKD (n = 65)	*p*
Age (years)	56.00 (47.00, 60.00)	56.00 (46.00, 60.00)	56.00 (47.00, 61.00)	ns
Male (n, %)	119, 66.1%	68, 59.1%	51, 78.5%	0.008
Duration of T2D (years)	4.79 (1.00, 10.72)	4.00 (1.00, 10.16)	5.00 (2.04, 13.87)	ns
BMI (kg/m^2^)	25.01 (22.95, 27.52)	24.52 (22.32, 26.91)	25.65 (23.92, 28.03)	0.025
Hypertension (n, %)	72, 40%	37, 32.2%	35, 53.8%	0.004
SBP (mmHg)	134.06 ± 17.76	130.97 ± 16.20	139.54 ± 19.14	0.002
DBP (mmHg)	84.51 ± 10.59	82.33 ± 9.20	88.37 ± 11.81	0.001
FBG (mmol/L)	8.51 (6.88, 10.90)	8.51 (6.93, 10.90)	8.40 (6.84, 10.77)	ns
HbA1c (%)	8.30 (7.00, 10.23)	8.30 (7.00, 10.33)	8.30 (7.10, 10.07)	ns
Hb (g/L)	144.18 ± 17.21	143.67 ± 17.31	145.03 ± 17.14	ns
Albumin (g/L)	45.50 (41.35, 48.90)	46.90 (42.20, 49.30)	44.25 (39.78, 47.48)	0.007
TC (mmol/L)	4.66 (3.88, 5.40)	4.70 (3.98, 5.46)	4.63 (3.65, 5.26)	ns
TG (mmol/L)	1.69 (1.10, 2.64)	1.54 (1.03, 2.36)	2.02 (1.19, 2.82)	0.044
HDL (mmol/L)	1.05 (0.91, 1.27)	1.08 (0.95, 1.33)	1.00 (0.88, 1.19)	0.010
LDL (mmol/L)	2.97 (2.33, 3.63)	3.04 (2.38, 3.70)	2.81 (2.23, 3.34)	ns
Creatinine (μmol/L)	66.00 (54.35, 75.55)	63.80 (52.60, 72.00)	71.70 (59.50, 99.90)	<0.001
eGFR (ml/min/1.73 m^2^)	101.77 (92.47, 109.48)	101.96 (96.51, 109.62)	97.37 (69.05, 108.38)	0.005
UACR ≥ 30 mg/g (n, %)	64, 35.6%	0	64, 98.5%	–
SIL-18 (pg/ml)	163.62 (117.63, 230.97)	166.76 (122.62, 231.96)	150.17 (109.05, 218.35)	ns
UIL-18 (pg/mg UCr)	140.82 (95.29, 227.40)	128.28 (82.67, 204.14)	193.24 (102.78, 257.81)	0.005
Cf-PWV (m/s)	8.30 (7.03, 9.70)	7.90 (6.80, 9.20)	9.20 (7.50, 10.25)	<0.001
Cr-PWV (m/s)	9.45 (8.43, 10.30)	9.30 (8.40, 10.20)	9.60 (8.60, 10.75)	ns

BMI, body mass index; cf-PWV, carotid–femoral pulse wave velocity; cr-PWV, carotid–radial pulse wave velocity; DBP, diastolic blood pressure; eGFR, estimated glomerular filtration rate; FBG, fast blood glucose; HDL, high-density lipoprotein cholesterol; Hb, hemoglobin; HbA1c, hemoglobin A1c; LDL, low-density lipoprotein cholesterol; SBP, systolic blood pressure; SIL-18, serum interleukin-18; TC, total cholesterol; TG, triglyceride; T2D, type 2 diabetes; UACR, urinary albumin-to-creatinine ratio; UCr, urinary creatinine; UIL-18, urinary interleukin-18.

According to the presence or absence of DKD, as described by UACR ≥ 30 mg/g and/or eGFR < 60 ml/min/1.73 m^2^, the proportion of male sex, BMI, systolic blood pressure (SBP), diastolic blood pressure (DBP), triglyceride, and serum creatinine were all significantly higher in the group of diabetic subjects with DKD than in the group without DKD. Subjects with DKD had significantly lower levels of albumin and high-density lipoprotein (HDL) compared with subjects without DKD. Interestingly, the urinary levels of IL-18 and cf-PWV were significantly elevated in subjects with DKD compared with those without DKD [UIL-18 193.24 (102.78, 257.81) vs. 128.28 (82.67, 204.14) pg/mg UCr, *p* = 0.005; cf-PWV 9.20 (7.50, 10.25) vs. 7.90 (6.80, 9.20), *p* < 0.001], whereas the serum IL-18 levels and cr-PWV were not different in the two groups.

By univariate linear regression analysis in **Table 2**, we found significant correlations between urinary IL-18 and age (*r* = 0.224, *p* = 0.002), male sex (*r* = −0.253, *p* = 0.001), duration of T2D (*r* = 0.259, *p* < 0.001), SBP (*r* = 0.228, *p* = 0.002), hemoglobin (*r* = −0.239, *p* = 0.002), albumin (*r* = −0.305, *p* < 0.001), eGFR (*r* = −0.237, *p* < 0.001), UACR ≥ 30 mg/g (*r* = 0.224, *p* = 0.003), and serum IL-18 (*r* = 0.219, *p* = 0.004) in participants with T2D. However, we found no significant correlation between urinary IL-18 and BMI, DBP, fast blood glucose, HbA1c, total cholesterol, triglyceride, HDL, low-density lipoprotein (LDL), or serum creatinine in participants with T2D. In the meantime, urinary IL-18 correlates with hemoglobin (*r* = −0.218, *p* = 0.026), albumin (*r* = −0.466, *p* < 0.001), eGFR (*r* = −0.393, *p* = 0.001), and serum IL-18 (*r* = 0.448, *p* < 0.001) in diabetic subjects with DKD. On the other hand, urinary IL-18 correlates with age (*r* = 0.209, *p* = 0.025), male sex (*r* = −0.35, *p* < 0.001), hemoglobin (*r* = −0.297, *p* = 0.003), and serum creatinine (*r* = −0.278, *p* = 0.003) in diabetic subjects without DKD.

**Table 2:** Univariate analysis of relationship between logarithmic serum or urinary IL-18 levels and characteristics of type 2 diabetes.

	Total (n = 180)				T2D − DKD (n = 115)				T2D + DKD (n = 65)			
	UIL-18 ^a^		SIL-18 ^a^		UIL-18 ^a^		SIL-18 ^a^		UIL-18 ^a^		SIL-18 ^a^	
	*r*	*p*	*r*	*p*	*r*	*p*	*r*	*p*	*r*	*p*	*r*	*p*
Age	0.224	0.002	0.128	ns.	0.209	0.025	0.088	ns.	0.239	ns.	0.245	ns.
Male	−0.253	0.001	0.020	ns.	−0.350	<0.001	0.056	ns.	−0.170	ns.	−0.010	ns.
Duration of T2D	0.259	<0.001	−0.110	ns.	0.171	ns.	−0.146	ns.	0.216	ns.	−0.086	ns.
BMI	0.044	ns.	0.137	ns.	0.044	ns.	0.285	0.003	−0.124	ns.	−0.065	ns.
Hypertension	0.170	0.023	0.105	ns.	0.128	ns.	0.114	ns.	0.125	ns.	0.119	ns.
SBP	0.228	0.002	0.072	ns.	0.189	0.043	0.019	ns.	0.186	ns.	0.173	ns.
DBP	0.138	ns.	0.055	ns.	0.088	ns.	0.097	ns.	0.059	ns.	0.035	ns.
FBG	0.009	ns.	0.054	ns.	−0.028	ns.	0.200	0.038	0.107	ns.	−0.224	ns.
HbA1c	0.047	ns.	−0.061	ns.	0.011	ns.	0.095	ns.	0.117	ns.	−0.373	0.003
Hb	−0.239	0.002	0.070	ns.	−0.297	0.003	0.071	ns.	−0.218	0.026	0.079	ns.
Albumin	−0.305	<0.001	0.288	<0.001	−0.175	ns.	0.346	<0.001	−0.466	<0.001	0.151	ns.
TC	0.028	ns.	0.157	0.042	0.025	ns.	0.110	ns.	0.049	ns.	0.209	ns.
TG	0.064	ns.	0.179	0.020	0.011	ns.	0.177	ns.	0.059	ns.	0.181	ns.
HDL	−0.093	ns.	−0.070	ns.	−0.072	ns.	−0.074	ns.	−0.026	ns.	−0.120	ns.
LDL	−0.026	ns.	0.190	0.014	−0.038	ns.	0.160	ns.	0.038	ns.	0.219	ns.
Creatinine	−0.037	ns	0.121	ns	−0.278	0.003	0.135	ns.	0.246	0.048	0.206	ns.
eGFR	−0.237	<0.001	−0.172	0.025	−0.094	ns.	−0.162	ns.	−0.393	0.001	−0.255	0.047
UACR ≥ 30 mg/g	0.224	0.003	−0.079	ns.	–	–	–	–	–	–	–	–
UIL-18 ^a^	1	–	0.219	0.004	1	–	0.111	ns.	1	–	0.448	<0.001
SIL-18 ^a^	0.219	0.004	1	–	0.111	ns.	1	–	0.448	<0.001	1	–
cf-PWV	0.309	<0.001	0.080	ns.	0.164	ns.	−0.004	ns.	0.418	<0.001	0.245	ns.
cr-PWV	−0.044	ns.	−0.072	ns.	−0.002	ns.	−0.118	ns.	−0.192	ns.	0.027	ns.

a, Urinary and serum IL-18 levels were analyzed as naturally logarithmically transformed values.

BMI, body mass index; cr-PWV, carotid–radial pulse wave velocity; DBP, diastolic blood pressure; eGFR, estimated glomerular filtration rate; FBG, fast blood glucose; HDL, high-density lipoprotein cholesterol; Hb, hemoglobin; HbA1c, hemoglobin A1c; LDL, low-density lipoprotein cholesterol; SBP, systolic blood pressure; SIL-18, serum interleukin-18; TC, total cholesterol; TG, triglyceride; T2D, type 2 diabetes; UACR, urinary albumin-to-creatinine ratio; UIL-18, urinary interleukin-18. ns, no significance.

We performed univariate analysis of the relationships between the parameters of arterial stiffness and the IL-18 levels in patients with type 2 diabetes (**Table 2**). The urinary IL-18 levels correlated positively with cf-PWV (*r* = 0.309, *p* < 0.001); however, we found no significant correlation between urinary IL-18 and cr-PWV in participants with T2D. Moreover, we only found significant correlation between the urinary IL-18 levels and cf-PWV (*r* = 0.418, *p* < 0.001) in diabetic subjects with DKD. We found no significant correlation between urinary IL-18 and cf-PWV in diabetic subjects without DKD. Nevertheless, we found no significant correlation between serum IL-18 and cf-PWV or cr-PWV in participants with T2D, diabetic subjects with DKD, or diabetic subjects without DKD.

To further clarify the link between urinary IL-18 and severity of arterial stiffness, we performed multiple regression analysis (**Table 3**), the association between urinary IL-18 and cf-PWV remained significant when controlling for age and gender (model 1) and additionally controlling for traditional arterial stiffness risk factors (model 2). Moreover, the association was little attenuated when further controlling for UACR and eGFR (model 3). Of note, although serum IL-18 had significant correlations with urinary IL-18 (*r* = 0.219, *p* = 0.004), none of such association was significant when urinary IL-18 and the traditional cardiovascular risk factors were simultaneously included in the model (model 3).

**Table 3:** Multivariable linear regression analysis of relationship between logarithmic urinary IL-18 and cf-PWV.

Variables	Model 1		Model 2		Model 3	
	β	*p*	β	*p*	β	*p*
UIL-18 ^a^	0.098	<0.001	0.077	0.005	0.062	0.037
Age	0.007	<0.001	0.006	0.002	0.004	0.028
Male	0.020	0.551	0.072	0.056	0.035	0.415
BMI			0.001	0.837	0.001	0.963
Duration of T2D			0.006	0.017	0.006	0.028
Hypertension			0.130	<0.001	0.098	0.007
Hb			−0.002	0.041	−0.002	0.122
Albumin			0.001	0.843	0.003	0.418
TC			0.027	0.019	0.012	0.388
HDL			0.027	0.649	0.054	0.400
eGFR					−0.001	0.389
UACR ≥ 30 mg/g					0.096	0.012
SIL-18 ^a^					0.026	0.499

eGFR, estimated glomerular filtration rate; FBG, fast blood glucose; Hb, hemoglobin; SIL-18, serum interleukin-18; TC, total cholesterol; T2D, type 2 diabetes; UACR, urinary albumin-to-creatinine ratio; UIL-18, urinary interleukin-18.

a, Urinary and serum IL-18 levels were analyzed as naturally logarithmically transformed values.

Model 1: Age and gender. Model 2: Model 1 and traditional arterial stiffness risk factors (BMI, duration of T2D, hypertension, Hb, albumin, TC, and HDL). Model 3: Model 2 and UACR, eGFR, and serum IL-18.

Given the association between urinary IL-18 and cf-PWV, the median cf-PWV was compared across the median of the urinary IL-18 levels. cf-PWV was greater in the higher group of urinary IL-18 than in the lower group (**Table 4**). Moreover, the differences persisted when adjusting traditional atherosclerotic risk factors and the presence of DKD (UACR ≥ 30 mg/g and/or eGFR < 60 ml/min/1.73 m^2^).

**Table 4:** Median of cf-PWV for subjects with T2D grouped according to the urinary levels of IL-18.

	UIL-18 (pg/mg UCr)		
	≤ 140.82	> 140.82	*p*
cf-PWV (m/s) 95%CI	8.050 (7.634, 8.466)	9.046 (8.629, 9.462)	0.001
cf-PWV (m/s) 95%CI ^a^	8.142 (7.719, 8.565)	8.914 (8.561, 9.311)	0.009
cf-PWV (m/s) 95%CI ^b^	8.475 (8.036, 8.915)	9.064 (8.679, 9.449)	0.046

cf-PWV, carotid–femoral pulse wave velocity; UCr, urinary creatinine; UIL-18, urinary interleukin-18.

a, Controlling for age and gender.

b, Additionally controlling for traditional arterial stiffness risk factors (BMI, duration of T2D, hypertension, Hb, albumin, TC, and HDL), and the presence of DKD (UACR ≥ 30 mg/g and/or eGFR < 60 ml/min/1.73 m^2^).

## Discussion

In the present study, we have found in subjects with T2D that the urinary IL-18 levels are associated with increased arterial stiffness as evaluated by cf-PWV. Moreover, the association was independent of the traditional arterial stiffness risk factors and the presence of UACR and eGFR. On the other hand, no significant correlation between serum IL-18 and cf-PWV was found in subjects with T2D. This is the first study that demonstrates the associations between the IL-18 levels and arterial stiffness in patients with T2D, with UACR and eGFR taken into account.

In current study, we found that the urinary IL-18 levels were higher in the group of diabetic subjects with DKD than in the group without DKD. This finding is approximately in line with those studies, suggesting that the higher level of urinary IL-18 has been a promise marker of kidney tubule injury (11, 12, 16). Of note, the positive correlation between the urinary IL-18 levels and cf-PWV was observed only in subjects with DKD, indicating that patients with diabetes with kidney involvement probably have a higher risk factor of CVD, and it was previously reported that subjects with DKD have the highest cardiovascular mortality compared to both patients with T2D without DKD and subjects without diabetes (17). Whether urinary IL-18 is associated with mortality or CVD requires further studies to clarify (18, 19).

The serum IL-18 levels are associated with albuminuria and atherosclerosis in patients with T2D (13). Subjects with elevated serum IL-18 were prone to develop T2D (20). However, elevated serum IL-18 was not associated with a higher risk for primary cardiovascular events in total diabetic population or diabetic subjects with renal dysfunction (21). In accordance with these controversial, we found no significant correlation between serum IL-18 and cf-PWV in participants with T2D, diabetic subjects with DKD, or diabetic subjects without DKD.

We have found that the higher IL-18 levels are associated with greater cf-PWV, suggesting link with arterial stiffness. When controlling for age and gender, urinary IL-18 was significantly associated with cf-PWV, and the association was independent of traditional arterial stiffness risk factors (22). The association remains significant when further controlled for UACR and eGFR, suggesting that the association is independent of kidney function markers. To further demonstrate the associations between IL-18 and arterial stiffness, mean cf-PWV was compared between the higher and lower groups of urinary IL-18. cf-PWV was greater in patients with higher urinary IL-18 than those with lower urinary IL-18, and the difference persisted when adjusting the traditional arterial stiffness risk factors. The differences between IL-18 and cf-PWV were not virtually modified when the presence of DKD was considered, further supporting the link between IL-18 and arterial stiffness.

Urinary IL-18 is produced within the kidney tissue in response to injury and inflammation (12). Urinary IL-18 is also expressed and secreted by macrophages in kidney diseases (23). The correlation between urine molecule markers and arterial stiffness may be explained by the following factors: 1. chronic kidney disease is a well-known risk factor for CVD, which is also a risk factor for arterial stiffness; 2. previous studies suggested that changes in markers of kidney injury often reflect renal tissue lesions and impaired renal function, which may affect the cardiovascular system and arterial stiffness through water, acid–base balance, electrolyte and mineral metabolism, endocrine, etc.; 3. urine markers reflect not only kidney disease but also the severity of systemic diseases, including diabetes, which is itself an important cardiovascular risk; 4. urine markers are likely to systematically reflect the extent of systemic lesions, that is, kidney lesions are likely to coexist with other organs in the body, including the heart, blood vessels, liver, and lung. These lesions probably progress together. Further studies may investigate the kidney-related mechanism on the development and progression of arterial stiffness in patients with diabetes and will help understand the reliability of urinary markers as specific biomarkers of CVD.

There are some limitations for the current study. First, we cannot currently determine the causal relationships between the IL-18 levels and greater arterial stiffness because of the cross-sectional design. Second, this study included substantial number of patients with T2D on medications, requiring studies to separate the effects of such medications. Furthermore, the source of urinary IL-18 in patients with T2D is not known. The elevated IL-18 levels in diabetic kidney tissue or infiltrated macrophage in the kidney may be responsible. Further experimental studies are needed to elucidate these questions.

In conclusion, we have demonstrated an association between the higher urinary IL-18 level and greater cf-PWV, suggesting the link between IL-18 and arterial stiffness in patients with T2D. This finding might offer a clue to understand the role of IL-18 in the development of CVDs.

## Data availability statement

The original contributions presented in the study are included in the article/supplementary material. Further inquiries can be directed to the corresponding authors.

## Ethics statement

The studies involving human participants were reviewed and approved by Institutional ethical committee of Nanjing Medical University. The patients/participants provided their written informed consent to participate in this study.

## Author contributions

Concept the study: JY and YZ; data acquisition: CS, AH, XW, and XZ; data analysis/interpretation: CS, LW, LJ, and YZ; statistical analyses: LJ, JY, and YZ; drafting the work and revising: JY and YZ. All authors contributed to the article and approved the submitted version.

## Funding

Natural Science Foundation of Jiangsu province: General program BK20201497 to YZ, and National Natural Science Foundation of China: General program 81873618 to JY.

## Acknowledgments

The authors thank the investigators, staff, and trial participants for dedication and commitment to the trial.

## Conflict of interest

The authors declare that the research was conducted in the absence of any commercial or financial relationships that could be construed as a potential conflict of interest.

## Publisher’s note

All claims expressed in this article are solely those of the authors and do not necessarily represent those of their affiliated organizations, or those of the publisher, the editors and the reviewers. Any product that may be evaluated in this article, or claim that may be made by its manufacturer, is not guaranteed or endorsed by the publisher.
